# Current research status of third-generation sequencing technology in thalassemia detection

**DOI:** 10.3389/fped.2025.1705599

**Published:** 2026-01-13

**Authors:** Fenglin Zhu, Yunli Lai, Sheng He

**Affiliations:** 1 Graduate School, Guangxi University of Chinese Medicine, Nanning, China; 2 Guangxi Clinical Medical Research Center for Birth Defects, Guangxi Key Laboratory of Reproductive Health and Birth Defects Prevention and Control, Guangxi Zhuang Autonomous Region Women and Children Care Hospital, Nanning, China

**Keywords:** clinical applications, genetic testing, long-read sequencing, nanopore sequencing, single-molecule real-time sequencing, thalassemia, third-generation sequencing

## Abstract

Thalassemia is a hereditary hemolytic disorder primarily caused by defects in the hemoglobin genes, which impede the synthesis of hemoglobin peptide chains. This disease is mainly classified into two types: α and β. Currently, there is no effective treatment available that can completely cure this disease. The conventional screening techniques for thalassemia currently used in clinical practice have significant shortcomings, posing risks of missed diagnoses and misdiagnoses. As a molecular detection technology that has emerged in recent years, third-generation sequencing can specifically address the shortcomings of conventional detection methods, enhance the positive detection rate for novel thalassemia variants, and demonstrate broad application prospects. However, it remains in the stage of technical exploration and refinement. This review aims to systematically organize and thoroughly analyze relevant research literature on the application of third-generation sequencing technology in thalassemia detection. It seeks to comprehensively understand the current status of utilization of this technology in thalassemia research, thereby fully leveraging its technical advantages to support the prevention, control, and management of thalassemia.

## 1 Introduction

Thalassemia was first described by Cooley and Lee in 1925. The disease was initially identified among populations along the Mediterranean coast, hence its name (1). It is also known as “thalassemia or hemoglobin synthesis disorder anemia,” with its causative factors related to human hemoglobin genes (2, 3). As a monogenic disorder, thalassemia follows the pattern of autosomal recessive inheritance (4). The α-globin gene cluster is located on chromosome 16 at position 16p13.3, while the β-globin gene cluster is located on chromosome 11 at position 11p15.3 (5, 6). Based on the different types of hemoglobin gene deletions, thalassemia can be classified into four subtypes: α, β, δ, and βδ. Among thalassemia patients, α and β types are the most common (7). From the perspectives of genotype and clinical manifestations, thalassemia can be classified into mild, intermediate, and severe forms. Clinically, mild (quiescent) patients typically exhibit no obvious symptoms or only mild symptoms, with most cases detected incidentally during physical examinations through thalassemia gene testing; intermediate patients present with typical hemoglobinopathy-related manifestations; severe patients may face risks of disability or death, posing a serious threat to health and life (8). Current research findings indicate that approximately 1%–5% of the global population carries thalassemia-related globin gene mutations (9); these genetic mutations primarily result from defects in hemoglobin synthesis and are prevalent in regions surrounding the Mediterranean Sea, North Africa, the Middle East, the Indian subcontinent, Southeast Asia, and southern China (10). In China, thalassemia is more prevalent in southern provinces such as Guangdong, Guangxi, Yunnan, Guizhou, and Hainan (11). In southern China, the rate of prevalence of the thalassemia gene defect ranges from 2.5% to 20% (12). With the intensification of global population mobility and the continuous advancement of science and technology, rare mutation forms within the *HBA1/2* and *HBB* genes have gradually been identified (13). The inherent complexity of genes and the intricate relationship between genotype and phenotype pose significant challenges to accurately diagnose carriers and patients (14).

From an economic burden perspective, a survey in Hunan, China, indicates that the average expenditure for non-surgical patients in 2023 was $16,005.60; for surgical patients, this figure increased significantly, averaging approximately $68,374.80 per case (15). Currently, the treatment of moderate-to-severe thalassemia presents significant challenges, with no specific curative therapies available. Conventional blood transfusion therapy provides only temporary symptom relief. A novel therapeutic approach aimed at correcting the genetic defect—involving the use of the lentiviral vector GLOBE to introduce functional genes into hematopoietic stem cells followed by intramedullary transplantation—is currently undergoing Phase I/II clinical trials. Its safety and efficacy require further validation (16). Recent studies have found that hematopoietic stem cell transplantation is one of the core therapeutic approaches for treating diseases such as thalassemia (17, 18). This condition is particularly common among children aged 0–5 years who are ill (19). Casgevy, a CRISPR-Cas9 gene-editing therapy for thalassemia, received approval in 2023 (20). In 2025, Furong Laboratory achieved multiple groundbreaking medical breakthroughs, including successfully conducting the world's first gene-editing treatment for thalassemia (21); in addition, HIF-2α inhibitors hold promise as a novel therapeutic approach for β-thalassemia (22). However, due to the generally high mortality rates associated with severe α- and β-thalassemias, the current global consensus strategy for thalassemia prevention and control relies on preconception and prenatal genetic screening to achieve the goal of healthy births (23).

The diagnosis of thalassemia requires a three-step process: initial screening, biochemical analysis, and confirmatory testing. Initial screening is based on MCV <80 fL and/or mean corpuscular hemoglobin <26 pg (24); Ferritin testing is then performed to rule out iron deficiency. Subsequent biochemical analyses, including Hb electrophoresis, are conducted sequentially, followed by confirmatory genetic testing using methods such as Gap-polymerase chain reaction (PCR) and PCR-based reverse dot blot (PCR-RDB). Although these methods are considered the gold standard for thalassemia screening, they present challenges such as cumbersome procedures, labor-intensive processes, and high costs (25). According to https://globin.bx.psu.edu/hbvar/menu.html, there are currently 1,907 types of human hemoglobin variations and thalassemia mutations in the database. The presence of compounding abnormal genotypes or modifier genes may further complicate the interpretation of thalassemia diagnoses; simultaneously, it compromises the diagnostic accuracy for rare mutations (26). Therefore, the development of novel DNA molecular diagnostic technologies is essential.

Next-generation sequencing (NGS), also referred to as high-throughput sequencing, represents a significant advancement in DNA sequencing technology. Its primary strength lies in its ability to perform massive parallel sequencing, allowing for the simultaneous processing of hundreds of millions of DNA fragments. This capability facilitates the rapid and cost-effective completion of large-scale sequencing projects, including whole-genome sequencing. The typical NGS workflow involves library preparation, template amplification, and sequencing-by-synthesis. By leveraging its high-throughput advantages, NGS has emerged as an indispensable tool in genomics, transcriptomics, and related research domains (27). It can simultaneously detect gene deletions and SNVs/indels (28), expanding testing coverage, while reducing reliance on routine testing. It also offers the advantages of simple sample collection and accurate results (29). However, due to various constraints (as given in Table 1), this technology cannot yet independently handle the screening and diagnosis of thalassemia and still requires supplementary support from Gap-PCR and Sanger sequencing (30). Notably, third-generation sequencing (TGS) has been applied to thalassemia gene testing in recent years (31), with its advantages positioning it as an alternative to traditional techniques; it demonstrates significant potential in the detection of this disease. This paper will proceed as follows:

**Table 1:** Advantages and limitations of various detection technologies.

Detection technology	Advantages	Shortcomings
Gap-PCR, PCR-RDB (28, 31, 100, 115)	Simple, low-cost	Only common thalassemia genotypes can be detected; primers designed near functional genes; near functional genes; missed diagnoses and misdiagnoses.
MLPA (94, 116–120)	Detectable rare deletions or duplications within the α- or β-globin genes; capable of identifying duplication and deletion phenomena; utilizing only several dozen probes.	Determining the hemoglobin beta-thalassemia genotype in specific scenarios (where deletions and duplications coexist) is challenging. It is impossible to localize unknown deletions/duplications within specific regions or detect unknown mutations, rendering the process complex, time-consuming, and costly.
Sanger (31, 121)	The gold standard for diagnosing Mendelian disorders is primarily applicable to the detection of known SNVs, indels, and known deletions.	Determining whether a mutation is cis or trans presents considerable difficulty; detecting large-scale deletions or duplications is also rather challenging.
NGS (27–29, 36, 106, 120, 122–126)	High-throughput, simultaneous detection of multiple genetic variants expands testing scope and reduces reliance on conventional methods, while offering straightforward sample collection and high testing accuracy.	The core shortcomings include short read lengths, low throughput, high costs, and limited applicability. Furthermore, overall sequencing accuracy is poor (prone to errors in specific regions and inaccurate copy number analysis), error rates are high, coverage is low, and it frequently leads to false positives or false negatives in detecting complex variations.
WGS (127–129)	Read length ranges from several hundred bp to several Mb; high precision. Comprehensiveness and freedom from *a priori* assumptions.	Drawbacks include high implementation costs, complex data analysis, and insufficient sensitivity for detecting low-frequency variants.
SMRT (28, 42, 106)	Featuring extended read lengths, high accuracy, and unbiased detection, this technology efficiently identifies hemoglobin variants to support independent sequencing. It offers real-time analysis, portability, high throughput, low cost, and rapid turnaround, making it suitable for diverse applications.	Sequencing technology faces constraints in widespread adoption because of high costs, limited accuracy, complex data processing, and the intricate relationship between genotypes and phenotypes.
ONT (33–35, 55, 130)		

SNVs, single nucleotide variants; indels, insertions/deletions; Gap-PCR, gap polymerase chain reaction; PCR-RDB, polymerase chain reaction-restriction digestion and blotting; MLPA, multiplex ligation-dependent probe amplification; sanger, chain termination method; NGS, next-generation sequencing; WGS, whole-genome sequencing; SMRT, single molecule, real time; ONT, Oxford Nanopore Technologies.

## 2 Principles and characteristics of TGS

**Figure 1:**
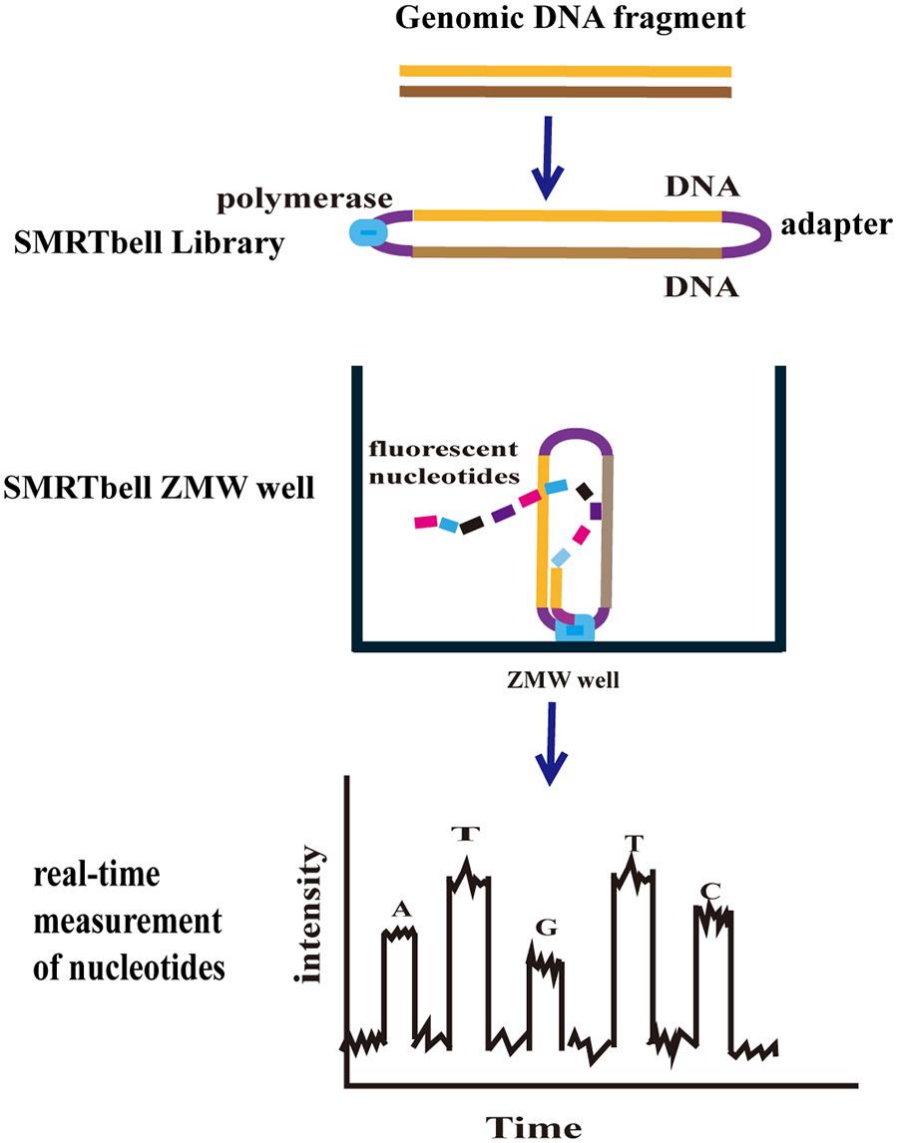
A partial workflow diagram based on single-molecule real-time sequencing. Adapted with permission from Hassan S, Bahar R, Johan MF, et al. Next-generation sequencing (NGS) and third-generation sequencing (TGS) for the diagnosis of Thalassemia. *Diagnostics (Basel)*. (2023) 13(3):373. Published 2023 Jan 19. doi:10.3390/diagnostics13030373. Copyright: © 2023 Author. Licensed by MDPI, Basel, Switzerland. This work is made available under the terms of the Creative Commons Attribution License (CC BY) (https://creativecommons.org/licenses/by/4.0/) for open access and distribution.

**Figure 2:**
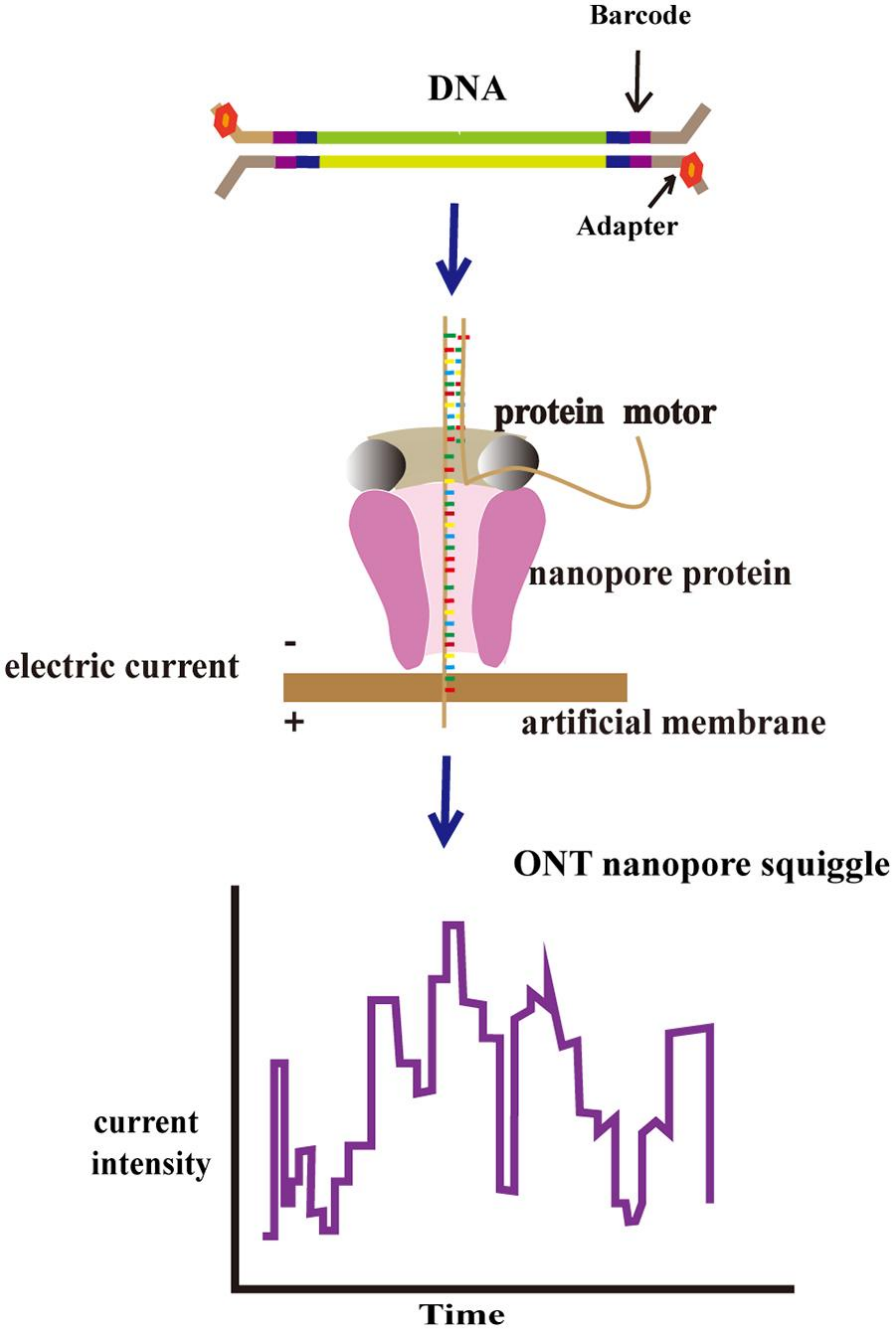
A schematic diagram of a partial workflow based on nanopores. Adapted with permission from Hassan S, Bahar R, Johan MF, et al. Next-generation sequencing (NGS) and third-generation sequencing (TGS) for the diagnosis of Thalassemia. *Diagnostics (Basel)*. (2023) 13(3):373. Published 2023 Jan 19. doi:10.3390/diagnostics13030373. Copyright: © 2023 Author. Licensed by MDPI, Basel, Switzerland. This work is made available under the terms of the Creative Commons Attribution License (CC BY) (https://creativecommons.org/licenses/by/4.0/) for open access and distribution.

TGS represents a major innovation in the field of genome sequencing, offering a new approach for deciphering complex genomic structural variations (32, 33). The two primary TGS platforms currently in use are Pacific Biosciences’ (PacBio) Single-Molecule Real-Time (SMRT) sequencing and Oxford Nanopore Technologies (ONT). SMRT employs single-molecule real-time sequencing principles, utilizing a strategy of sequencing during synthesis. Four fluorescent labels mark four dNTPs, and during base-pairing chain synthesis and extension, the incorporation of different bases emits distinct fluorescence signals. The DNA base sequence is determined based on the type and duration of these fluorescence signals (Figure 1). The ONT principle involves DNA molecules passing through nanopores, where different nucleotides in the sequence cause perturbations in the current flowing through the pore. The sequencer records the electrical signals generated during DNA passage through the pore and then translates the specific sequence of electrical signals into a nucleotide sequence (34) (Figure 2). ONT offers advantages such as rapid library preparation, real-time sequencing data analysis, a multiplex long PCR, and improved alignment capabilities (35). SMRT sequencing, with its advantages in long-read sequencing (LRS), has been employed for comprehensive and valuable thalassemia testing (36, 37). In addition, ONT offers a range of instruments flexibly tailored to different throughput requirements, from the portable MinION to the ultra-high-throughput PromethION. Its sequencing chips are also reusable, significantly reducing experimental costs (38). With continuous advancements in molecular diagnostic technologies, thalassemia detection methods have evolved from traditional hematological parameter analysis to the application of high-throughput techniques. Different detection approaches possess distinct advantages, while also exhibiting corresponding limitations. In recent years, ongoing research has focused on refining the strengths of existing technologies, while simultaneously addressing their specific shortcomings. This paper reviews relevant literature to summarize commonly used, established, or emerging diagnostic technologies in molecular thalassemia testing, along with the advantages and limitations of third-generation sequencing technologies published in recent years, as detailed in Table 1.

Although TGS currently incurs relatively high costs, research by Huang et al. (39) confirms its irreplaceable role in detecting rare thalassemia variants. As sequencing throughput continues to increase and costs gradually decrease, TGS shows great promise for widespread application in genetic disease screening and clinical diagnosis in the future. Based on an analysis of relevant literature (40–45) and inquiries on official platforms related to ONT (see note to Table 2), the following summary has been generated: the total cost of sequencing projects is primarily composed of equipment acquisition, per-run expenses, and investments in professional personnel. Equipment prices vary significantly among manufacturers. For instance, PacBio’s product line ranges from the early PacBio RS, which costs approximately $800,000, to the more affordable Sequel model launched in 2015, priced around $350,000. This is followed by the higher-priced next-generation Sequel II and Sequel IIe models. Similarly, ONT provides a range of options that includes the portable and low-cost MinION, available for a few thousand dollars, as well as mid-to-high-throughput systems like GridION and PromethION, which can cost tens to hundreds of thousands of dollars. In addition to equipment costs, data production expenses also vary considerably across platforms. When utilizing HiFi mode, PacBio Sequel II and Sequel IIe achieve a cost per gigabase (Gb) between $15 and $30. Within the ONT platform, the per-Gb costs for MinION and GridION range widely from $20 to $200, while the high-throughput PromethION significantly lowers costs to $5–15 per Gb, making it more suitable for large-scale projects. Professional labor costs are another critical factor to consider. The entire workflow—from sample preparation and library construction to sequencing—requires the expertise of molecular biology technicians. Moreover, the analysis and interpretation of extensive sequencing data, particularly long-read data, necessitates the involvement of bioinformatics specialists. These personnel expenditures should be carefully planned in advance within the project budget. Furthermore, innovations in third-generation sequencing technology provide new avenues for diagnosing thalassemia. We evaluate its cost-effectiveness in comparison with conventional diagnostic methods (refer to Table 2 for details).

**Table 2:** Cost comparison of third-generation sequencing technology and selected common traditional thalassemia diagnostic methods

Technical name	Instrument cost	Consumables cost	Single-run cost	Single-base cost (USD/million bases)	Data processing costs	Applicable Scenarios	Literature/website source
Sanger sequencing	Tens of thousands of dollars	Relatively high	Hundreds of dollars	1–10	Low	Small-scale precision sequencing	Tan et al. (131), https://www.thermofisher.com/us/en/home/life-science/sequencing/sanger-sequencing.html
Next-generation sequencing	Hundreds of thousands of dollars	Moderate	Hundreds of dollars	0.05–0.15	Moderate	Large-scale genome sequencing	Tan et al. (131), https://www.illumina.com/techniques/sequencing.html
Third-generation sequencing	Hundreds of thousands to millions of dollars	High	Several thousand to tens of thousands of dollars	0.33–1.00	High	Complex genome analysis, long-read sequencing	https://www.pacb.com/ https://nanoporetech.com/ https://pdf.dfcfw.com/pdf/H3_AP202503171644442323_1.pdf https://pdf.dfcfw.com/pdf/H3_AP202308211595387393_1.pdf van Dijk El et al. (132)
Gap-PCR	None (no reliance on conventional PCR equipment)	Low (primer synthesis and PCR reagents)	Tens of dollars	Not applicable (non-sequencing technology)	Low	Small-scale gene fragment detection	https://www.thermofisher.com/us/en/home/life-science/pcr/gap-pcr.html
PCR-RDB		Moderate (primer synthesis, PCR reagents, and gel electrophoresis)	Tens of dollars			Rapid specific gene detection	https://www.thermofisher.com/us/en/home/life-science/pcr/pcr-rdb.html
Agarose gel electrophoresis	None (standard laboratory equipment)	Low (gel material)	A few dollars			Nucleic acid isolation and purification	https://www.thermofisher.com/us/en/home/life-science/pcr/agarose-gel-electrophoresis.html
PCR		Low (primer synthesis and PCR reagents)	Tens of dollars			Nucleic acid amplification	https://www.thermofisher.com/us/en/home/life-science/pcr.html

Inquiries on official platforms related to ONT: https://www.pacb.com/sequencing-technology; https://www.bio-itworld.com/news/2015/09/25/pacbio-to-ship-350k-sequel-sequencer-in-2016; https://nanoporetech.com/products; Search “PacBio sequencing cost” or “HiFi cost per Gb” in https://www.pacb.com/sequencing-technology/; PacBio Sample Preparation Guide (https://www.pacb.com/support/documentation/); or ONT Library Preparation Protocol (https://community.nanoporetech.com/protocols).

Low: ≤$7.07/sample (primarily consumable costs, no/minimal data processing costs). Medium: $7.21–$28.28/sample (core consumable costs, low-to-moderate data processing costs). Relatively high: $28.42–$707/sample (sequencing technology-driven, includes significant data processing costs). High: >$707/sample (complex sequencing protocols with both high consumable and data processing costs).

## 3 The core advantages of TGS in thalassemia gene testing

### 3.1 Fundamental breakthrough in technical performance

Since 2021, TGS has been widely applied in the detection of thalassemia, with a significant increase in the number of related research reports. A systematic search was conducted in the authoritative database PubMed (https://pubmed.ncbi.nlm.nih.gov/; as of October 2025) using four sets of keywords: The first group comprised “thalassemia” and “third-generation sequencing,” the second group included “thalassemia” and “single-molecule real-time sequencing,” the third group consisted of “thalassemia” and “nanopore sequencing,” and the fourth group contained “thalassemia” and “long-Read Sequencing.” The qualifying search results comprised 41, 23 (with nine duplicates overlapping the first group), 8, and 12 articles (with 21 duplicates overlapping the first three groups). After deduplication, a total of 84 relevant publications were obtained, with research reported between 2021 and 2025. Among these 84 reports, 77 originated from China (see Tables 3–6 for details).

**Table 3:** The status of third-generation nanopore sequencing technology (ONT) for the detection of thalassemia from 2021 to 2025 (eight articles).

Reference and year	Location	Number of subject tests	Core contributions of ONT technology
Zhan et al., 2023 (133)	Guangxi, China	–	The screening and diagnosis of TGS in thalassemia
Huang et al., 2023 (34)	Guangdong, China	150	The strategy of multiplex long PCR is an efficient strategy for library preparation
Jiang et al., 2021 (103)	Guangdong, China	13 Families	Targeted nanopore sequencing, combined with the RHDO analysis, is feasible for NIPT for β-thalassemia
Erlich et al., 2024 (105)	Chandigarh, India; Oakland, CA, USA	121 Families	To develop a non-invasive prenatal test for beta-hemoglobinopathies based on analyzing maternal plasma
Chin et al., 2024 (66)	Singapore	1 Family	Provided clarity and enabled family planning for the proband and her family
Wei et al., 2025 (55)	Guangdong, China	36	ONT has the potential for comprehensive variant detection in the diagnosis of thalassemia and other genetic diseases
Zeng et al., 2025 (69)	Guangdong, China	1 Case report	Use family analysis or third-generation sequencing technology to elucidate complex mutation associations
Liu et al., 2021 (102)	Beijing, China	4	TGS on the Oxford Nanopore is a potential alternative and universal method for PGT

**Table 4:** Detection of thalassemia using third-generation PacBio (SMRT) platforms from 2021 to 2025 (40 articles)

Reference and year	Location	Number of subject test	Core contributions of PacBio (SMRT)
Ling et al., 2023 (134)	Guangxi, China	–	Review and comparison of different sequencing technologies
Xu et al., 2024 (63)	Hunan, China	504	A comparative analysis of TGS and traditional PCR methods for CATSA
Liu et al., 2023 (85)	Hunan, China	3,033	Evaluating a new third-generation sequencing-based method called CATSA
Long et al., 2022 (46)	Guangxi, China	4	A new tool for detecting complex variants in the α-thalassemia gene
Zhuang et al., 2023 (54)	Fujian, China	70	A new method for identifying α- and β-thalassemia alleles based on comprehensive analysis using TGS
Lou et al., 2023 (50)	Guangdong, China	19,932	CATSA is a more comprehensive and accurate method than traditional PCR
Ren et al., 2023 (111)	Guangdong, China	20	TGS diagnosis has great potential for diagnosing suspected cases of rare thalassemia in children, especially TDT
Li et al., 2024 (135)	Guangdong, China	–	TGS has significant advantages in supplementing other genetic diagnostic methods
Wu et al., 2022 (56)	Guizhou, China	176	Exploring the application of TGS in genetic diagnosis and prenatal genetic screening for thalassemia
Xu et al., 2023 (74)	Hubei, China	Two families	A new α-thalassemia deletion has been discovered
Tang et al., 2025 (96)	Guangdong, China	200	A comprehensive approach to screening and diagnosis of rare thalassemia
Huang et al., 2024 (51)	Hainan, China	1,122	TGS is a more accurate and reliable method for screening carriers of thalassemia
Peng et al., 2022 (58)	Sichuan, China	100	Further validation of TGS as a promising method for rare thalassemia gene detection
Traisrisilp et al., 2024 (136)	Chiang Mai, Thailand	–	For thalassemia testing, TGS can provide additional genetic diagnosis
Hassan et al., 2023 (117)	Malaysia	–	Diagnosis of thalassemia has shifted from traditional DNA analysis in laboratories to NGS or TGS analysis
Li et al., 2022 (47)	Guangxi, China	32	Long-read TGS is a reliable and efficient approach for accurate detection of HKαα thalassemia
Zhuang et al., 2024 (84)	Fujian, China	6,174	Enriching the spectrum of thalassemia provides valuable data and highlights the clinical application value of TGS
Long et al., 2024 (62)	Guangxi, China	2,000	CATSA has become the most powerful technical support for the three-tier prevention and control strategy for thalassemia
Huang et al., 2025 (39)	Fujian, China	119	The efficacy of TGS and thalassemia gene diagnostic kits in identifying Thal gene mutations
Zhuang et al., 2024 (83)	Fujian, China	Two families	Two rare variants of Hb Jilin and Hb Beijing were discovered.
Ning et al., 2023 (48).	Guangxi, China	1,845	SMRT technology improves diagnostic accuracy and positive detection rate for thalassemia
Zhou et al., 2024 (114)	Sichuan, China	One case report	Identification of a new complex variant, expanding the spectrum of thalassemia gene variants
Ye et al., 2025 (60)	Guangdong, China	1,020	T-LRS captured 20 genes/loci in 1,020 β-thalassemia patients
Liang et al., 2023 (52)	Guangxi, China	One case report	TGS identification of patients with double heterozygosity −α4.2Ⅰ/−α4.2Ⅱ
Chen et al., 2023 (137)	Yunnan, China	One case report	Identification of a new α-globin gene triplicate by TGS
Zhong et al., 2022 (78)	Guangdong, China	One case report	TGS technology is a powerful tool for detecting thalassemia breakpoints
Jiang et al., 2024 (80)	Guangdong, China	One case report	Discovery of a new α-globin gene cluster deletion, accurately described by TGS as a 16.8 kb deletion
Zhuang et al., 2024 (88)	Fujian, China	Four families	Combining Hb capillary electrophoresis with TGS can effectively improve the screening and diagnosis of Hb Lepore
Zhang et al., 2025 (98)	Guizhou, China	22,480	Targeted TGS analysis of thalassemia gene mutations to assess the mutation spectrum in this population
Li et al., 2023 (75)	Guangxi, China	One family	A 107 kb deletion causing α-thalassemia is reported for the first time worldwide
Chen et al., 2022 (101)	Guangdong, China	One case report	TGS detected a 4.9 kb deletion in the *HBB* gene
Zhuang et al., 2025 (67)	Fujian, China	Two families	Identification of a rare HBB: c.316-90 a >g variant
Zeng et al., 2025 (95)	Guangdong, China	One case report	Family history analysis or third-generation sequencing technology to clarify complex mutation associations
Guo et al., 2024 (81)	Guangxi, China	One case report	TGS discovers a new type of α-thalassemia in newborns—145 kb
Zhong et al., 2022 (92)	Guangdong, China	One case report	A novel 7.2 kb deletion was identified for the first time in the *HBB* gene
Meenakumari et al., 2025 (138)	Tamil Nadu, India	–	Various molecular technologies, established gene-editing methods, epigenetic mechanisms, and artificial intelligence for the management of thalassemia
Liu et al., 2025 (70)	Sichuan, China	One case report	TGS can detect mutations more accurately and identify rare genotypes and rearrangements of the α-globin gene cluster
Liang et al., 2021 (37)	Hunan, China	1,159	TGS combines remote PCR to provide a universal solution for screening carriers of thalassemia genes
Zeng et al., 2025 (69)	Guangxi, China	55,281	Understanding the genotypes and distribution of thalassemia in the Guibei region
Li et al., 2025 (68)	Guangxi, China	65	Molecular diagnosis and clinical analysis of a 1,357 bp deletion in the Guangxi cohort

T-LRS, targeted long-read sequencing; TDT, suspected transfusion-dependent thalassemia; TGS, third-generation sequencing; CATSA, comprehensive analysis of thalassemia alleles.

**Table 5:** The current situation of single-molecule real-time sequencing (SMRT) for thalassemia detection from 2021 to 2025 (23 articles) (duplication of nine references with Table 3)

Reference and year	Location	Number of subject tests	Core contributions of SMRT
Jiang et al., 2022 (100)	Guangdong, China	Nine patients and their family members	Demonstrated significant advantages in detecting α-globin gene duplications and rare deletions and determining cis- or trans-configurations
Luo et al., 2022 (28)	Guangxi, China	434	Carriers of rare *HBB* gene variants such as c.-100G>A, c.-136C>G, c.315+5G>c, c.380T>G, and c.-81A>c; 16 rare HBA gene variants; 14 cases of rare α-globin gene deletions or triplications
Zhang et al., 2023 (86)	Fujian, China	33	All HKαα alleles detected by SMRT were consistent with conventional techniques. Two variants not identified by conventional methods were detected in β-thalassemia
Xu et al., 2024 (57)	Guangdong, China	3	Three rare genetic variants were identified: αααanti-3.7, Hb Lepore–Boston–Washington, and Hb anti-Lepore P-India
Zhong et al., 2022 (78)	Guangdong, China	One case report	A novel 15.8 kb deletion was identified within the *HBA* gene, with its precise location determined for the first time (16p13.3, Chr16:163886-179768, GRch38/hg38)
Luo et al., 2022 (31)	Guangxi, China	4	Identification of rare thalassemia variants not detected by four conventional diagnostic methods
Zhou et al., 2023 (53)	Hainan, China	23	Fusion mutations were successfully detected; diagnosis corrected in two “pure” heterozygotes: one being a compound heterozygote with anti-3.7 triploidy and the other a homozygote
Xu et al., 2023 (74)	Fujian, China	One family	First report of a 91.5 kb deletion in the HS-40 region of the α-globin gene cluster (αα) FJ identified in a Chinese family
Wu et al, 2025 (139)	Hunan, China	Expert consensus	Exploring the scope of application, operational procedures, and limitations of single-molecule real-time sequencing in clinical thalassemia gene testing
Bao et al., 2022 (99)	Guangdong, China	One familyc	The α3.7III subtype was identified for the first time in Chinese patients; its phenotypic effects were analyzed through lineage analysis
Qin et al., 2023 (90)	Guangdong, China	2 cases	Two cases of Hb Q-Thailand heterozygosity unrelated to the (−α4.2/) deletion allele were identified for the first time
Bao et al., 2023 (76)	Guangxi, China Guangdong, China	4	Four novel deletions were identified at the α-globin locus using SMRT sequencing technology for the first time
Chen et al., 2023 (87)	Guangxi, China	23,709	Classification of the 15 zones delineated by hemoglobin electrophoresis. One novel hemoglobin variant, Hb Anti-Lepore Liuzhou, and nine rare hemoglobin variants were identified within zones Z1–Z12
Jiang et al., 2023 (94)	Guangdong, China	1,316	Accurate genetic diagnosis of the proband using molecular biology techniques is crucial for prenatal diagnosis of rare thalassemia
Rangan et al., 2021 (49)	Minnesota	Four cases	Evaluated four known complex β-globin gene cluster indel types and demonstrated high concordance with previously reported data
Wu et al., 2022 (106)	Guangdong, China	Three families	Establishing a preimplantation genetic diagnosis method for β-thalassemia based on long-read sequencing
Bao et al., 2022 (82)	Guangdong, China	One proband	Identification of a novel 5 kb deletion; Expansion of the pedigree for deletion-type β-thalassemia
Yuan et al., 2023 (77)	Hunan, China	One proband	Identification of a 14.9 kb large deletion at the α-globin gene locus (hg38 chr16:168,803–183,737), which disrupts the pre-exon and the HBA1 and HBA2 genes of the mother
Xu et al., 2025 (97)	Guangdong, China	One proband	CNVplex technology identified a novel α-globin gene deletion, with the size of the deletion determined via customized TGS being 146 kb.
Ning et al., 2024 (104)	Guangxi, China	One family	First discovery of compound heterozygosity for the −11.1 kb and the −SEA alleles
Huang et al., 2025 (89)	Guangxi, China	33,958	Reporting a novel mutation involving a 7.412 kb repeat in HBB and HBD, resulting in a new Hb anti-Lepore Laibin
Xu et al., 2025 (72)	Jiangxi, China	One proband	Described a novel quadruple-copy structure of the gamma globin gene within HBG2 and reported the molecular characteristics of a complex thalassemia involving mutations in the *HBG1*/*HBG2*, *HBD*, and *HBB* genes
Zhong et al., 2024 (79)	Huizhou, China	One case report	A novel case of Hb Bart's hydrops fetus was identified, exhibiting typical α₀-thalassemia (*−^SEA^*) deletion and a novel 45.2 kb deletion encompassing three functional hemoglobin genes

**Table 6:** The current situation of long-read sequencing for detecting thalassemia from 2021 to 2025 (12 articles) (21 articles were duplicated with the first three groups)

Reference and year	Location	Number of subject tests	Core contributions of SMRT
Lee et al., 2025 (107)	Taiwan, China	One case report	This study aimed to explore the feasibility of third-generation LRS in the genetic diagnosis of such a rare condition in a prenatal setting
Feng et al., 2023 (108)	Guangdong, China	One family lineage	An LRS-based approach directly identified that the exact deletion region was chr16: 48,642–132,584, which was located in the α-globin upstream regulatory elements and named (αα)^JM^ after Jiangmen city
Li et al., 2025 (91)	Shanghai, China	2,926 participants	This study aimed to evaluate the clinical application of long-read sequencing for screening carriers of four complex diseases: spinal muscular atrophy, α/β-thalassemia, 21-hydroxylase deficiency, and fragile X syndrome
Zhou et al., 2025 (109)	Beijing, China	244 Participants	This study identified two novel deletion variants in the HBA gene, expanding the genotype spectrum of α-thalassemia.
Toledo et al., 2023 (59)	USA	One editorial	A review article focusing on the clinical application of long-read sequencing technology in prenatal diagnosis of α- and β-thalassemia
Liang et al., 2023 (110)	Hunan, China.	278 High-risk pregnancies involving the fetuses of thalassemia carriers	CATSA represents a more comprehensive and accurate approach that potentially enables more informed genetic counseling and improved clinical outcomes compared with PCR-based methods
Ye et al., 2025 (60)	Guangdong, China	1,020 Participants	The largest T-LRS study conducted on patients with β-thalassemia provides crucial evidence for precise clinical diagnosis and phasing analysis of the globin gene cluster haplotypes
Oliveira et al., 2023 (112)	USA	Two female and newborn twins	A novel deletion was reported in two females and newborn twins, involving the ɛ, Aγ, Gγ, and ψβ loci, while the LCR, δ region, and β region remained intact
Shi et al., 2025 (61)	Guangxi, China	115 Participants	Verification of the Accuracy and Clinical Utility of TLRPGT-α-thal
Zhong et al., 2025 (73)	Guangdong, China	A Chinese boy with β-thalassemia intermedia phenotype and his family	Novel structural variants were identified within the α-globin gene cluster, confirming that long-read sequencing is the preferred method for detecting rare novel structural variants
Zhuang et al., 2023 (93)	Fujian, China	One family lineage	This paper described a novel δ/β-globin gene deletion identified through long-read sequencing technology
Shao et al., 2023 (113)	Yunnan, China	One case report	With this novel large deletion found in the HBB gene in China, we expanded the genotype spectrum of β-thalassemia and showed the advantages of long-read sequencing (LRS) for comprehensive and precise detection of thalassemia variants

#### 3.1.1 Comprehensive and precise typing

Long et al. (46) conducted an analysis of four abnormal samples using TGS, successfully identifying complex genotypes within the α-globin gene cluster, including one case with the variant *αα^anti3.7^α^anti3.7^α^17.2^*. This work offers valuable insights for the detection of similar variants. However, it is important to note that the study's small sample size of only four abnormal cases may restrict the generalizability of the findings, therefore, further validation with a larger sample size is warranted. Base insertion errors and false negatives in TGS for α-thalassemia deletion detection can compromise the reliability of prenatal diagnosis and genetic counseling by interfering with genotyping and haplotype phasing, thereby reducing the accuracy of genetic risk assessment. Li et al. (47) examined 32 patients suspected of possessing the *HKαα* genotype and demonstrated that TGS can accurately identify the *HKαα* allele, outperforming traditional methods such as gap-PCR. Furthermore, TGS effectively differentiates between the genotypes *HKαα*/*αα*, *HKαα*/*−α^3.7^*, *HKαα*/*−α^4.2^*, and *HKαα/–^SEA^*. Notably, this detection method does not necessitate family analysis, making it suitable for broader application in clinical practice. Ning et al. (48) employed SMRT technology to identify relevant genetic variants associated with thalassemia, including one rare variant of *HBA2* and six variants of *HBB: -α^3.7^/HBA2:c.300+34G>A*, *HBB:c.316–45G>C/β^N^*, *HBB:c.315+180T>C/β^N^*, and *HBB:c.316–179A>C/β^N^*. This study provides a molecular foundation for the prevention and control of thalassemia in the Yulin region. Rangan et al. (49) utilized SMRT to systematically assess four known types of complex insertion/deletion mutations in the β-globin gene cluster. Their findings illustrate that long-read sequencing can effectively identify these complex variations. Nevertheless, the stringent sample preparation requirements pose a significant limitation to routine clinical applications, highlighting the need for workflow optimization to facilitate large-scale testing. Lou et al. (50) randomly selected subjects with both positive and negative hemoglobin test results for Comprehensive Allele Typing by Single Nucleotide Amplification (CATSA) detection; this method achieved a 5.42% increase in amplification yield and identified a novel deletion in the α-globin gene cluster (chr16:171262–202032). Consequently, the study calculated the carrier frequencies for α, β, and α-/β-thalassemia at 5.62%, 3.85%, and 0.93%, respectively. However, the random sampling approach may not adequately represent the entire population, and there may be statistical errors in the carrier frequency estimates. Future research should involve a comprehensive analysis that incorporates data from additional regions. Huang et al. (51) simultaneously performed second-generation and third-generation sequencing on 1,122 individuals in Hainan Province; TGS revealed 2.28% more results than second-generation sequencing, detecting 746 variants compared with 17. Furthermore, TGS demonstrated greater accuracy and reliability for screening thalassemia carriers when compared with second-generation sequencing.

#### 3.1.2 Misjudgments correction and clarification capabilities

Multiple studies have demonstrated that TGS technology can effectively rectify misclassifications in thalassemia genotyping performed using conventional methods. Liang et al. (52) showed that conventional techniques often misclassify Hb Q-Thailand compound heterozygotes as homozygotes for a 4.2 kb deletion; in contrast, TGS can accurately identify both the 4.2 kb deletion and the Hb Q-Thailand compound heterozygosity (*−α^4.2^*/*−α^4.2−Q−Thailand^*, *−α^4.2Ⅰ^*/*−α^4.2Ⅱ^*). Zhou et al. (53) employed SMRT technology to perform a comprehensive analysis of 23 carriers, effectively correcting genotyping errors arising from the misclassification of compound heterozygotes and homozygotes as pure heterozygotes by conventional NGS; their findings confirm that SMRT is a superior method for detecting α-globin fusion genes. Zhuang et al. (54) conducted a blinded study demonstrating that CATSA technology can enhance the detection rate of rare α- and β-globin gene variants by 7.14%, effectively identifying variants that are often overlooked by conventional methods.

#### 3.1.3 High sensitivity and reliability

Wei et al. (55) addressed the absence of a performance evaluation system for ONT prior to its clinical application by developing a thalassemia mutation classification method capable of automatically parsing, presenting, and identifying mutations. Utilizing ONT to analyze 36 samples, the team accurately identified 19 single nucleotide variants (SNVs), six deletions, and two duplication variants. The results demonstrated 100% consistency with known information, with no false positives or false negatives. With a detection limit of 3 ng/μL, this study underscores the reliable potential of the method as a comprehensive variant detection tool for diagnosing related diseases. Wu et al. (56) demonstrated that TGS achieved a concordance rate of 99.43% with traditional genotyping methods, successfully detected rare variants that could not be identified by PCR-RDB, which relies on predesigned probes for known mutations, such as the β-thalassemia *−*50 [G>A] mutation, and accurately assessed the risk of severe pregnancy complications in homozygous carrier couples. These findings highlight the suitability of TGS for prenatal screening in high-prevalence regions. Xu et al. (57) precisely identified three rare gene variants using SMRT sequencing, demonstrating that this technology can detect fusion gene breakpoints with greater accuracy than traditional methods, which are often prone to false negatives. Peng et al. (58) reported that TGS detected 10 additional rare variants in 100 suspected cases, including the first identification of the *−α^3.7III^* variant in the Chinese population. Their findings confirm that traditional methods are prone to misdiagnosing rare variants. Toledo et al. (59) noted that although long-read sequencing technology may offer only limited improvements, its enhanced sensitivity and specificity are critical for the prenatal diagnosis of thalassemia. These advantages help mitigate the substantial risks associated with misdiagnosis in key reproductive decisions. Ye et al. (60) achieved highly accurate diagnoses of β-thalassemia using T-LRS technology in a single test; this approach not only identified pathogenic mutations with 100% accuracy but also detected novel haplotypes and rare modifier mutations that cannot be identified by conventional methods, thereby significantly enhancing prognostic outcomes. These findings provide crucial evidence for personalized treatment. Shi et al. (61) established a preimplantation genetic testing (PGT) method for α-thalassemia based on targeted long-read sequencing; by leveraging its core capability to perform mutation and linkage analysis simultaneously in a single-tube reaction, this method demonstrated 100% accuracy through large-scale validation, underscoring its exceptional reliability.

#### 3.1.4 Clinical efficiency and practicality

Long et al. (62) employed capillary electrophoresis (CE), hotspot detection, TGS, and CATSA to screen for thalassemia variants in 2,000 neonatal samples. Their results demonstrated that CATSA achieved the highest detection rate, identifying 535 cases (26.75%) and covering multiple variant types. In addition, CATSA directly identified the cis–trans relationship of variants in three newborns, significantly reducing the diagnostic timeline. Compared with other techniques, CATSA offers distinct advantages and shows promise as a core technology for the three-tiered prevention and control of thalassemia. Li et al. (47) further demonstrated that CATSA facilitated variant genotyping without the need for family-based analysis, thus enhancing its suitability for clinical implementation. A comparative study by Xu et al. (63), although previous studies have reported that TGS on the PacBio platform can achieve detection costs of approximately $20 (64, 65), demonstrated that CATSA outperformed PCR technology in thalassemia gene analysis, despite its higher cost and limited applicability. The Sequel II platform is costly and requires bulk sample pooling for sequencing, restricting its application scope; therefore, developing a low-cost, low-throughput benchtop PacBio sequencing platform holds greater clinical feasibility. Currently, CATSA covers only the *HBA* and *HBB* genes and has been validated exclusively in hemoglobin-positive samples.

#### 3.1.5 Focus on the clinical analysis of specific mutation types

Chin et al. (66) reported a proband with elevated HbA2 levels and no pathogenic *HBB* variants, whose partner was a thalassemia carrier. Through targeted long-read sequencing with ONT, they identified a specific heterozygosity in the *KLF1* gene, which is associated with benign *HbA2* elevation, while excluding pathogenic *HBB* variants. This finding suggests that when routine thalassemia gene testing yields no abnormalities, it is essential to extend the analysis to include the regulatory gene *KLF1* to prevent missed diagnoses. Zhuang et al. (67) and Li et al. (68) conducted clinical and hematological phenotype analyses, along with molecular diagnostic evaluations of *HBB: c.316–90A>G* and a 1,357 bp deletion. Their accurate identification of molecular defects through phenotype–genotype correlation highlights the importance of integrating hematological parameters with genetic testing results for a comprehensive interpretation. This approach not only clarifies thalassemia carrier status in cases involving complex variants but also provides a foundation for genetic counseling.

### 3.2 Expansion of diagnostic capabilities

#### 3.2.1 Analysis of complex variants

Zeng et al. (69) identified a rare combination of β-thalassemia gene mutations *[β^−28(A>G)^* with *IVS-I-5(G>A)/β^CD 71/72(+A)^]* in the Chinese population using ONT, thereby filling a gap in the domestic mutation spectrum. This discovery underscores the unique advantages of long-read sequencing in elucidating complex allelic variations, particularly in addressing cis/trans positioning challenges that traditional Sanger sequencing often struggles to resolve. Furthermore, the study emphasizes that when Sanger sequencing fails to clarify complex mutations, family analysis or TGS should be employed to elucidate their genetic characteristics. In Liu et al.’s study (70), NGS revealed four tandem 3.7 kb repeats in α-globin gene cluster 1 and a specific heterozygous insertion–deletion (InDel) in *HBA1* gene cluster 2. Both the TGS method validated by their research and this NGS approach provided comprehensive coverage of the *HBA1* and *HBA2* genes. Notably, TGS demonstrated superior resolution in resolving chimeric variants within highly homologous regions (e.g., *HBA1/HBA2*) and accurately localizing breakpoints, enabling precise characterization of rare variants. Meanwhile, Luo et al. (28, 31) concentrated on key genes, including *HBA1*, *HBA2*, and *HBB*, and expanded primer usage to detect specific deletions. Although the increased number of primers contributed to higher costs (a methodological limitation), they successfully identified multiple rare variants and abnormal cases, including two 27,311 bp deletions in the α-globin gene cluster. The precise characterization of these large-scale deletions underscores the necessity of long-read sequencing for detecting atypical breakpoints and complex rearrangements, thereby clarifying the hematological phenotypes associated with certain variants and affirming the diagnostic value of SMRT technology. Li et al. (71) identified a 10.7 kb deletion (Chr16:154,355–165,114del;NG_000006.1:g.15218_25972del) and a homozygous point mutation (Chr16:169,854T>C; NG_000006.1:g.30717T>C; rs2258435) in this case. This finding highlights the importance of phasing point mutations and large deletions on the same allele for accurately assessing their compound heterozygous or homozygous status, as well as the corresponding phenotypic severity. Meanwhile, Xu et al. (72) identified novel structural variants involving *HBG1* and *HBG2* duplications through SMRT sequencing and first reported a new quadruplet-structured γ-globin gene {*HBG1/HBG2*[*^G^γ^A^γ/−158(C>T)^G^γ^G^γ^G^γ^A^γ*]}. This discovery not only reveals the complex patterns of *γ*-globin gene duplication but also underscores the indispensable role of long-read sequencing in resolving structural variations within highly repetitive, multicopy gene clusters. Zhong et al. (73) successfully identified a novel large-scale repeat (αααα*280*) within the α-globin gene cluster; utilizing long-read sequencing technology, they accurately determined both the precise size and the internal structure of this repeat sequence, enabling direct sequencing of the complete repeat unit. This methodology mitigated the errors commonly associated with NGS short-read assembly, providing strong evidence for the critical role of LRS in addressing complex genetic disease diagnoses.

#### 3.2.2 Structural defect detection

Research teams led by Xu et al. (74), Li et al. (75), Bao et al. (76), and Yuan et al. (77) independently identified multiple novel deletion variants within the α-hemoglobin gene, with lengths ranging from 10.3 to 107 kb. Notably, these studies documented the first reported a 107 kb deletion globally, alongside China's initial discoveries of 91.5 and 14.9 kb deletions. Several of these deletions affect critical disease-causing genes, thereby enriching the molecular variation landscape associated with α-thalassemia. Moreover, these studies confirmed that advanced technologies such as TGS, SMRT, and LRS outperformed traditional methods like multiplex ligation-dependent probe amplification (MLPA) in accurately locating deletion ranges and identifying rare or complex variants. Consequently, the authors recommended that prenatal diagnostic approaches for high-risk populations incorporate conventional testing in conjunction with SMRT screening for rare mutations. Zhong et al. (78, 79) identified a novel 15.8 kb deletion through SMRT sequencing, which also revealed a new case of Hb Bart's hydrops fetalis, precisely mapping a 45.2 kb deletion at the mutation site, thereby expanding the spectrum of α-thalassemia mutations. Jiang et al. (80) successfully characterized a new 16.8 kb deletion within the α-globin gene cluster using custom-designed MLPA probes, a multiplex long PCR, and TGS, associating this deletion with a homologous recombination event. Guo et al. (81) reported a significant α-thalassemia deletion exceeding 145 kb, named the Guigang deletion (–Guigang), in honor of the proband's hometown. Bao et al. (82) discovered and characterized a novel 5 kb deletion within the Chinese population, enhancing the understanding of deletion-type β-thalassemia and providing new insights for further investigations into the functional dynamics of the β-globin gene cluster.

#### 3.2.3 Rare variant screening

Huang et al. (34) developed a novel TGS method for detecting hemoglobinopathy variants using the ONT MinION platform; by preparing specific libraries through multiplex long-read PCR, they achieved a precise differentiation of target gene variants; their results from 158 thalassemia samples were fully consistent with known genotypes, offering valuable insights for advancing TGS technology. Zhuang et al. (83) employed TGS technology to identify two rare hemoglobin variants, Hb Jilin and Hb Beijing, in Fujian Province. In addition, their subsequent study (84) identified four thalassemia-associated variants in the Chinese population for the first time: *α^CD30(−GAG)^α* (the first report from Fujian), Hb Lepore–Boston–Washington, as well as two novel variants, *β^CD15 (TGG>TAG)^* and *β^IVS−II−761^*, and a *β*^0^-Philippine (approximately 45 kb deletion); in total, they detected 35 hemoglobin variants, including the two newly reported variants from Fujian, along with one compound variant. Liu et al. (85) utilized the CATSA method to identify eight variants in 49 individuals that were not detected by other techniques, including the first identification of Lepore Hb in Hunan Province. Zhang et al. (86) also applied CATSA to detect two β-thalassemia variants (*HBB:C.341T>A* Hete and *HBB:C.316-45g>C* Hete). Chen et al. (87) detected ten rare hemoglobin variants in the Z region of South China among 23,709 samples, including first reports in Asia and Guangxi, as well as one novel variant. Their findings confirmed the association of these variants with the occurrence of thalassemia, supplemented regional data, and provided crucial support for prenatal diagnosis. Zhuang et al. (88) studied δβ-thalassemia and identified novel Hb Lepore–Hong Kong variants, alongside rare deletion-type δβ-thalassemia variants. Utilizing TGS, they elucidated the mechanisms underlying these mutations and also discovered similar Hb Lepore–Boston–Washington variants in affected patients. Huang et al. (89) identified two novel hemoglobin variants, Hb Laibin (*HBA2:c.44T>C*) and Hb Anti-Lepore Laibin, enriching the hemoglobin gene mutation database with relevant cases. Qin et al. (90) were the first to diagnose two heterozygous cases of the rare Hb Q-Thailand variant using SMRT sequencing, validating the new genotype and confirming that Hb Q-Thailand was not necessarily associated with the (*−α^4.2^/*) allele. Li et al. (91) demonstrated that LRS identified a greater number of pathogenic variants than NGS in carrier screening for high-frequency genetic disorders such as thalassemia, achieving particularly high detection rates for α- and β-thalassemia. Zhong et al. (92) identified a novel 7.2 kb deletion in the HBB gene for the first time. In addition, Zhuang et al. (93) identified a novel 7.4 kb deletion (NG_000007.3:g.63511_70924del) in a Chinese family through long-read sequencing. This mutation led to a partial deletion of the *HBB* and *HBD* genes, resulting in the formation of δ–β fusion genes and contributing to δβ-thalassemia. Jiang et al. (94) reported a prenatal diagnosis rate of 3.19% (42 out of 1,316) for rare thalassemia variants; the most prevalent alleles associated with α- and β-thalassemia in the Chinese population are *^G^γ(^A^γδβ)0*, while the *–^THAI^* deletion is the most common in the Thai population.

In the context of regional epidemiology and rare variant exploration, Zeng et al. (95) analyzed data from over 50,000 patients in Guilin, elucidating the local distribution characteristics of thalassemia genotypes and providing valuable evidence for the development of regional prevention and control strategies. Tang et al. (96) applied TGS to 72 suspected cases in Zhongshan City, resulting in a 5.6% increase in thalassemia detection rates and reporting four novel rare deletion types of the *HBA* gene (*−11.1*, *−α^27.6^*, *−α^2.4^*, and *−α^21.9^*). Xu et al. (97) identified and validated a novel 146 kb deletion, confirming its pathogenic potential. In addition, Zhang et al. (98) employed TGS to create the first molecular map of thalassemia in Guizhou Province, identifying 12 abnormal hemoglobin genotypes along with multiple common variant types.

#### 3.2.4 “One-stop” solution

Bao et al. (99) employed a combination of detection technologies to identify the *−α^3.7 III^* subtype mutation for the first time in China, with SMRT technology playing a crucial role in genotyping validation throughout the process. Zhong et al. (78) and Jiang et al. (100) each utilized SMRT technology to identify novel gene deletion fragments and multiple rare mutations, confirming the technology's superior performance in detecting rare variants, gene duplications, deletions, and determining variant configurations. Zhong et al. (78) also discovered a novel 15.8 kb deletion. Chen et al. (101) systematically identified and validated both known and novel mutations in the *HBA* and *HGB* genes by integrating targeted detection with second- and third-generation sequencing technologies, leading to the discovery of a new 4.9 kb deletion in the *HBB* gene. Liang et al. (37) demonstrated that CATSA was a targeted detection method for thalassemia developed on a third-generation sequencing platform. Compared with traditional PCR techniques, NGS and Sanger sequencing, its core advantages lie in broad detection coverage, high precision in identifying genetic variants, outstanding efficacy in identifying large structural variants, and the elimination of the need for additional cross-validation experiments. Its simplified operational workflow provides a more efficient and reliable technical solution for molecular diagnosis in the field of thalassemia. It has detected 1,317 PCR-confirmed pathogenic variants and multiple rare variants without any false negatives in a cohort of 1,159 samples, thereby optimizing carrier identification and supporting its use for screening high-risk couples. In addition, targeted sequencing, combined with remote PCR, shows promise as a universal screening approach. Liu et al. (102) developed a novel PGT strategy based on ONT. By constructing approximately 5 kb large-fragment DNA libraries through multiple displacement amplification, this approach enables high-precision identification of large-fragment deletions and translocation breakpoints associated with α-thalassemia, while simultaneously achieving short tandem repeat (STR) linkage analysis and whole-chromosome/segmental aneuploidy screening. This method is particularly suitable for screening balanced translocation carriers and identifying deletion variants in PGT for monogenic disorders, offering the potential for automation and platform compatibility, which may allow more laboratories to perform cost-effective and efficient PGT testing. Notably, this study represents the first application of ONT in the field of PGT.

#### 3.2.5 Precise genotyping and non-invasive detection capabilities

Jiang et al. (103) conducted research on non-invasive prenatal testing (NIPT) by recruiting 13 families at high risk for β-thalassemia. They performed haplotype genotyping using two library sizes (10 and 20 kb), with the 20 kb library achieving complete genotyping. As a result, the fetal risk status was accurately determined for 12 families, demonstrating that longer read libraries enhance haplotype reconstruction completeness and improve diagnostic success rates. Ning et al. (104) were the first to report that compound heterozygosity for the −11.1 kb and the *–^SEA^* alleles resulted in hydrops fetalis in patients with Hb Bart's. Erlich et al. (105) utilized two core technologies to enhance fetal genotype prediction accuracy: in silico size selection (ISS) enrichment of fetal cell-free DNA (cfDNA) with short read retention, combined with 2.2 kb amplicon sequencing using Nanopore MinION to determine *HBB* haplotypes, followed by alignment with NextGENe LR software. Their results indicated that ISS, in conjunction with *HBB* haplotype analysis, enabled accurate prediction, whereas predictions based solely on variant read proportions were inadequate, underscoring the necessity of haplotype typing for fetal genotype inference. Wu et al. (106) developed a long-read sequencing-based PGT-M method that facilitated β-thalassemia embryo haplotype linkage analysis without the need for parental DNA samples, addressing the issue of allelic dropout in genetic markers. This approach offers a novel strategy for preimplantation genetic testing independent of parental contributions. Lee et al. (107) identified an exceedingly rare genomic deletion (approximately 27,411 bp) shared by both the fetus and the father through long-read sequencing analysis. The precise size and breakpoints of this deletion could be determined only by using this technology, highlighting the unique advantages of long-read sequencing in resolving complex structural variations. Feng et al. (108) detected a *–^SEA^* deletion in the father using conventional methods, with no variation observed in the mother; long-read sequencing confirmed that the deletion spanned the 48,642–132,584 region on chromosome 16 (upstream of the *α*-globin locus), a mutation termed the Jiangmen mutation [*(αα)^JM^*]. This finding exemplifies the technique's capability to uncover atypical deletion-type thalassemias that might be overlooked by standard approaches. Zhou et al. (109) employed CATSA to analyze thalassemia genes in 244 pregnant women with abnormal blood test results in North China, and their findings revealed that 16.39% of the participants carried thalassemia mutations, including rare cases and 44 distinct variant types. Among these, the most prevalent variants were *−α^3.7^*, *–^SEA^*, and *HBB:c.316-197C>T.* Notably, CATSA technology identified eight additional variants compared with conventional methods, demonstrating its critical value in the precise genotyping of 22.50% of carriers. This study also identified two novel deletions in the *HBA* gene, supporting enhanced thalassemia screening and expanding population coverage in northern China. These results underscore the clinical significance of long-read sequencing in improving the detection of thalassemia mutations and rare variants. Liang ' (110) detected 206 fetal variants using CATSA, surpassing PCR detection methods, which identified 191 cases, thereby representing a 7.9% increase in detection rate. This advanced method corrected phenotypic predictions for eight fetuses and identified α-globin triploidy in two cases, adjusting the phenotype classification from β-thalassemia characteristics to an intermediate type, thus altering prognosis assessments. This demonstrates that long-read sequencing can rectify misjudgments in traditional methods due to missing information, thereby optimizing genetic counseling and pregnancy management. Ren et al. (111) suggested that third-generation sequencing (TGS) holds significant potential for diagnosing suspected cases of rare thalassemia in children, particularly in the context of transfusion-dependent thalassemia. In Oliveira's study (112), newborn screening indicated a characteristic Hb A to Hb F expression ratio in both twins. A molecular analysis revealed a 32.6 kb deletion spanning the *HBE1* to *HBBP1* genes. This novel deletion resulted in an unreported form of ɛ*γ* thalassemia with a unique phenotype, thereby expanding the genotype–phenotype spectrum of thalassemia and highlighting the utility of long-read sequencing in identifying novel pathogenic mechanisms. Shao et al. (113) utilized CATSA to precisely identify a novel 10.8 kb deletion in the *HBB* gene that causes thalassemia. This pathogenic variant was first detected in the patient and his paternal brother, with its presence subsequently corroborated by hemoglobin testing results. Zhou et al. (114) reported the case of an 18-year-old Chinese woman who was found to have a novel complex variant, *α^Hb Westmead^/α^Hb Westmead^/α^anti3.7^/−α^3.7^*, in association with a rare form of α-thalassemia.

## 4 Challenges and prospects in TGS for thalassemia detection

### 4.1 In terms of ONT

ONT technology is currently in the exploratory phase of research and development for thalassemia detection. Although it is a research hotspot, existing ONT literature indicates that relevant studies are scarce and predominantly focused on the β-thalassemia field. Library preparation is a core step in NGS and TGS applications. TGS must overcome the challenge of amplifying long templates through multiple rounds of long PCR, while ONT requires a dedicated SNP caller to enhance accuracy. Although ONT can effectively detect multiple variants of thalassemia, its performance evaluation and validation system remains unestablished—a critical prerequisite for clinical application and regulatory approval. In addition, molecular diagnosis of thalassemia is challenging due to the complex mutations in the *HBA* and *HBB* genes, the high GC content and high homology of the globin genes, and bottlenecks in mutation classification, all of which exacerbate diagnostic difficulties. The current ONT faces challenges such as high raw error rates and complex data processing. Future improvements can focus on enhancing accuracy and integrating bioinformatics tools to advance toward a vision of seamless, real-time, portable precision medicine. Subsequent efforts should expand research on α-thalassemia and deepen studies on β-thalassemia, leveraging the strengths of ONT to support thalassemia prevention, control, and clinical management while refining its genetic profile.

### 4.2 PacBio (SMRT) technology

The application of SMRT long-read sequencing technology in thalassemia detection faces several limitations. Its high testing costs, limited sequencing throughput, and stringent requirements for sample DNA integrity and quality, coupled with complex data analysis workflows that heavily rely on specialized bioinformatics expertise, and incomplete clinical validation systems and standardized operating procedures, constrain its large-scale clinical adoption. However, SMRT sequencing offers distinct advantages, enabling simultaneous detection of multiple variants such as complex structural alterations and point mutations in a single run. It also facilitates α-globin genotyping and mutation haplotype phasing, thereby achieving precise diagnosis. With declining sequencing costs, increased throughput, and the optimization of data analysis workflows toward automation and standardization, SMRT sequencing is poised to replace multitechnique screening strategies as the new standard for molecular diagnosis of thalassemia, thereby advancing genetic counseling and precision medicine.

## 5 Conclusion

As an emerging high-throughput molecular diagnostic technology, TGS is reshaping the genetic diagnosis pathway for thalassemia, driving its transformation from traditional isolated and fragmented testing methods toward an integrated, systematic precision diagnosis and treatment model. This technology not only significantly enhances the coverage and accuracy of mutation detection, establishing itself as an efficient and reliable option for clinical diagnosis, but also provides critical technical support for deeply analyzing the complex genetic background and molecular mechanisms of thalassemia. It lays a crucial foundation for ultimately conquering this disease.
